# Sequence Bundles: a novel method for visualising, discovering and exploring sequence motifs

**DOI:** 10.1186/1753-6561-8-S2-S8

**Published:** 2014-08-28

**Authors:** Marek Kultys, Lydia Nicholas, Roland Schwarz, Nick Goldman, James King

**Affiliations:** 1 Science Practice Ltd, London, 83-85 Paul Street, EC2A 4NQ, UK; 2 European Molecular Biology Laboratory, European Bioinformatics Institute, Wellcome Trust Genome Campus, Cambridge, Hinxton, CB10 1SD, UK

## Abstract

**Background:**

We introduce Sequence Bundles--a novel data visualisation method for representing multiple sequence alignments (MSAs). We identify and address key limitations of the existing bioinformatics data visualisation methods (i.e. the Sequence Logo) by enabling Sequence Bundles to give salient visual expression to sequence motifs and other data features, which would otherwise remain hidden.

**Methods:**

For the development of Sequence Bundles we employed research-led information design methodologies. Sequences are encoded as uninterrupted, semi-opaque lines plotted on a 2-dimensional reconfigurable grid. Each line represents a single sequence. The thickness and opacity of the stack at each residue in each position indicates the level of conservation and the lines' curved paths expose patterns in correlation and functionality. Several MSAs can be visualised in a composite image. The Sequence Bundles method is designed to favour a tangible, continuous and intuitive display of information.

**Results:**

We have developed a software demonstration application for generating a Sequence Bundles visualisation of MSAs provided for the BioVis 2013 redesign contest. A subsequent exploration of the visualised line patterns allowed for the discovery of a number of interesting features in the dataset. Reported features include the extreme conservation of sequences displaying a specific residue and bifurcations of the consensus sequence.

**Conclusions:**

Sequence Bundles is a novel method for visualisation of MSAs and the discovery of sequence motifs. It can aid in generating new insight and hypothesis making. Sequence Bundles is well disposed for future implementation as an interactive visual analytics software, which can complement existing visualisation tools.

## Background

Sequence Bundles is a novel method for collation, visual representation, exploration and analysis of multiple sequence alignment (MSA) data [1]. Since its development, this method has been used to visualise and expose a number of sequence motifs and data features in protein alignments. The Sequence Bundles method was presented at the IEEEVis 2013 conference in Atlanta, Georgia, where it was awarded the ex aequo honourable mention in the BioVis 2013 data redesign contest.

### Motivation

With the continuous development of ever more powerful methods for data collection and generation, we are faced with the challenge of not only making sense of this abundance of information, but also making good use of it. Modern computational methods for structuring data, finding patterns and querying databases address many of these challenges already. However, in many processes, the abilities intrinsic to human perception are still not matched by computers. Such processes include: rapidly recognising complex and non-obvious patterns; instant inferring, deducing and ad hoc hypothesis-forming; following sound and scientifically informed intuition. We aimed at capitalising on these human abilities and tried to bring sequence data analysis closer to human experience.

Our motivation in creating, developing, and putting Sequence Bundles to practical use was to allow for the discovery of hidden sequence motifs and other data features in a visualised dataset by direct manipulation and visual analysis of that data visualisation itself. Sequence Bundles is a visualisation method aimed at aiding scientific discovery by enabling the process of direct exploration where visualisation can be used as a sandbox for rapid testing of hypothesis, suppositions and even speculations about MSAs.

We also aimed at designing a visualisation method that would demonstrate potential for being relatively accessible to domain non-specific readers (e.g. prospective collaborators). By revealing more--more intuitively than existing MSA visualisation methods--the Sequence Bundles method is designed with the intent to be equally approachable and attractive to both practitioner and non-practitioner audience groups.

### Related work

With the current growth in the amount of biological data, its scale, variety and complexity, new strategies and tools for exploring this wealth of knowledge are required [2,3]. Moreover, in order for this knowledge to be understandable and usable for both expert and interdisciplinary audiences, it needs to be presented in accessible, transparent and intuitive ways.

In bioinformatics, a convention of the Sequence Logo has been developed [4] in order to enable the display of a range of MSA features in a single graphic: the consensus sequence, relative frequencies of residues at every position, the amount of information present at every position measured in bits, as well as significant locations in the input alignment. Further developments which build on the Sequence Logo method include inter alia: HMMLogo (giving visual representation to both emission and transition probabilities of Profile Hidden Markov Models--pHMMs) [5]; Seq2Logo (including other important information in the visual output, e.g. about the low number of observations) [6]; CodonLogo (a tool that allows for visual discrimination between patterns of codon and nucleotide conservation) [7]; and pLogo (visualising residue heights scaled relative to their statistical significance) [8]. All of these developments are in essence variations on the original Sequence Logo visualisation method by Schneider and Stephens [4] and even though they enhance the Logo visualisation by the addition of novel features, they also retain the Logo's inherent limitations.

Some kinds of information buried in MSAs cannot be easily exposed by either the Sequence Logo method, or any of its variations. When addressing those MSA features designers of visualisation tools need to rely on combining other methods [9] or--as in case of the Sequence Bundles--creating new ones.

### Objectives

In a series of interviews and workshops with bioinformaticians from the United Kingdom, United States and Poland (see the 'Acknowledgements' section), we identified a number of requirements that a successful MSA visualisation should support, as well as a number of limitations and redundant features of the existing Sequence Logo method that should be addressed. This led our design efforts towards the following objectives:

**1 -- **Although Sequence Logos are very effective in exposing the general consensus sequence, as well as amino acid distribution on each position, they also obscure patterns in the relationships between sites within the sequences. This results in very important information about residue correlation and non-obvious sequence affinity being removed completely from the visualisation. Our general goal was, therefore, to reintroduce this relational information to the visualisation in order to facilitate and assist visual exposure of sequence motifs.

**2 -- **Our scientific interviewees saw little benefit in showing the amount of information on each position, measured in Sequence Logos against the Y-axis and expressed in bits. In fact, some scientists were surprised to learn about that during the interview, as they had never used this measure before. Displaying the amount of information seemed to be addressed to a far more specialised user. Therefore, our aim was to remove this data from the Y-axis and repurpose the axis for the benefit of a larger and more interdisciplinary audience.

**3 -- **Some visualisation tools are well suited for showing details, while others favour a more global inspection. Residue statistical detail and localised sequence properties can be easily identified and described by using Sequence Logos (or even by inspecting parts of a MSA itself). However, the Logo method is of limited value when applied to datasets with longer sequences, because of its site-specific focus. Thus, our objective was to favour global inspection of datasets by designing a visualisation encoding which is capable of exposing macroscopic patterns and generating findings of sequence-wide significance.

**4 -- **A Sequence Logo hides important information about the total number of analysed sequences (this information exists in the length of a MSA itself) and their relative affinity (relative distance from each other on the phylogenetic tree). Consequently, our aim was to provide an indication of the sample size (number of sequences in a visualised MSA).

**5 -- **The Sequence Logo visualisation method is equally well equipped to display either DNA or protein MSAs. In fact, the Logo visualisation principles should be easily applied to any sequential dataset which can be formatted as a MSA. Our goal was to retain this universal scope of application.

In line with our motivation, and in order to address Sequence Logo limitations and other visualisation challenges identified during our research, we decided to abandon the convention of Sequence Logo and develop a completely new method for visualising MSA data, which we explain below. First in the 'Methods' section we outline iterative design methodologies employed in the project, followed by an explanation of the Sequence Bundles visual encoding and a summary of key departures from the Sequence Logo. Later, in the 'Results' section, we describe the extent to which Sequence Bundles has been developed and list a number of interesting data features exposed in the competition dataset by using our visualisation method. Finally, we conclude with a discussion around the interactive potential of the Sequence Bundles method, which can complement existing visualisation tools to expose what otherwise could remain hidden.

## Methods

### Design methods

We approach bioinformatics visualisation from the perspective of information design. Information design is a design discipline focused on 'defining, planning, and shaping of the contents of a message and the environments in which it is presented, with the intention of satisfying the information needs of the intended recipients' [10]. In our case the MSA is the contents of a message and the recipients are bioinformatics practitioners. Taking this approach and using methodologies and techniques practiced in the design world, we developed Sequence Bundles in the following research-led and iterative design process:

**1 -- **Desk research phase -- in which we conducted a multidisciplinary and multi-level literature review and acquired basic understanding of bioinformatics fundamentals;

**2 -- **Initial sketching phase -- in which we tried to produce Sequence Logos ourselves by using both fictional and real data. This enabled us to understand how exactly Sequence Logo visual encoding works, which features it exposes, and which it conceals;

**3 -- **External research phase -- in which we interviewed a number of molecular biology and bioinformatics experts to learn about their scientific work, their opinion on Sequence Logos and its strengths and limitations, as well as their reasons for which they decide to use or not to use the Logo in their practice;

**4 -- **Prototyping on paper and idea generating phase -- in which we brainstormed new concepts for sequence data representation, explored diverse strategies for visually encoding bioinformatics data, investigated ways in which Sequence Logos can be redesigned, and prototyped all our ideas in sketches, drawings and mock-ups;

**5 -- **Stimulus research and ideas refinements phase -- in which we consulted with bioinformatics experts presenting them our prototyped ideas once again to obtain detailed explanations of how selected approaches can function. For this phase we simulated visualisation outcomes with real small MSAs;

**6 -- **Prototyping in code phase -- in which we developed the Sequence Bundles demonstration application to generate actual visualisations of the BioVis 2013 redesign contest dataset, which helped in further refinements of the visual encoding;

**7 -- **Visual analysis and insight generation phase -- which emerged unplanned, when we started exploring and editing vector visualisations generated with the demonstration application. In this phase we discovered a number of features in the competition data, which were given salient expression by the Sequence Bundles visual encoding. We discuss some of these features in the 'Results' section.

**8 -- **Presentation and expert feedback phase -- took place at the IEEEVis 2013 conference in Atlanta, Georgia, where we presented the Sequence Bundles method and our findings to the BioVis 2013 contest jury and other experts in the field. We received valuable feedback regarding our developments thus far and discussed potential directions for future work.

### Visual encoding

**Figure 1:**
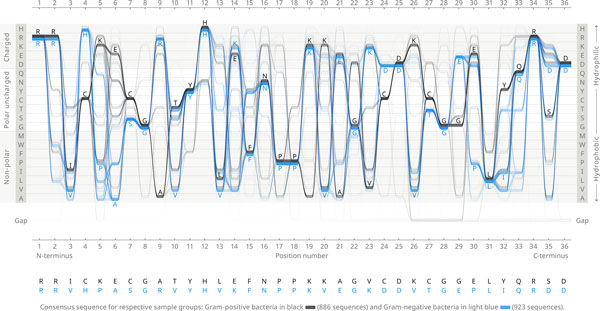
**Sequence Bundles comparing amino acid distribution and correlation in the AKL domain**. Bundled visualisation plots sequences as stacked lines against a Y-axis of letters arranged on a scale representing amino acid hydrophobicity. The lines' curved paths expose the conservation of residues by converging at matched positions. Their place relative to letters on the Y-axis exposes patterns in functionality. The consensus sequence is indicated. Lines representing two groups of organisms differ by colour: Gram-positive bacteria (black lines) and Gram-negative bacteria (blue lines). The visualisation is generated from a total of 1809 AKL protein sequences. The number of samples is: 923 Gram-negative sequences vs. 886 Gram-positives, which is in 100:96 ratio.

Figure 1. shows a Sequence Bundles visualisation of the BioVis 2013 redesign contest dataset [11]. The visualised MSA contains 1809 aligned sequences of the adenylate kinase lid (AKL) domain sampled from two groups of bacteria: Gram-positive (886 sequences labelled black) and Gram-negative (923 sequences labelled blue). Each sequence in the MSA is 36 positions long. All visualisations throughout the paper are based on this dataset provided for the contest entrants (see the 'Acknowledgements' section).

The Sequence Bundles method plots sequences as stacked lines against horizontal X-axis, which marks sequence base or residue numbers, and against vertical Y-axis, on which residues are arranged on a scale of their physicochemical properties (in Figure 1 it is the scale of amino acid hydrophobicity ordered after Wampler [12]) and marked with their letter symbols. A distinct Y-axis position is used for gap characters in the MSA. One line represents each protein sequence. Read from left to right, the line's precise shape plotted against both axes corresponds to the sequence of specific residues displayed on each subsequent site. This visual encoding of sequences combined with their meaningful vertical organisation allows for saliently exposing patterns in their properties and functionality, (e.g. when amino acid hydrophobicity defines the Y-axis, the more hydrophilic each sequence fragment is, the closer to the top of the chart it will appear; conversely, the more hydrophobic it is, the lower the line will be plotted).

In Figure 1 we contrast two families of bacteria by compositing two coloured sub-Bundles (Gram-positives are black and Gram-negatives are blue). Each sub-Bundle is created by plotting all lines representing individual sequences from the respective MSA and stacking them in sets of 10. In Figure 1, for the Gram-positive sub-Bundle all black lines displaying arginine (R) in position 1 will be arranged in stacks of 10 and overlaid at least 88 times. Lines are collated in the same order in which sequences reside in the MSA. Line thickness in Sequence Bundles is uniform and set to prevent white gaps from appearing between neighbouring lines; thereby a stack of many lines appear as bundled together. In order to enable the distinction between denser and less dense stacks, lines in Sequence Bundles are semi-transparent. In all figures in this paper line transparency is set to 98% (2% opacity) in normal blending mode to enable clear display of overlaying lines and motifs. Both the thickness and the opacity of the stack of lines at each letter in each position indicate the level of localised consensus between sequences. The general consensus sequence for each group of sequences compared in the MSA is also shown. Optimal line tangency in Sequence Bundles was selected in our iterative design process, providing reductions in visual clutter created by intersecting lines and improvements in perceptual clarity of the image.

Comparison of sub-Bundles in the composite Sequence Bundles visualisation is facilitated by the use of labelling by colour, as well as by plotting each group with a vertical offset relative to one another. The selection of black and saturated light blue colours in Figure 1 complies with the best practices of visual design [13], as it enables users with any kind of colour-blindness to discern each sub-Bundle, thus allowing an even greater range of users to comfortably work with Sequence Bundles.

### Key departures from sequence logos

The Sequence Bundles method was conceived as a redesign of the existing long-standing convention of Sequence Logos. However, the extent to which Sequence Bundles departed from the Logo qualifies it as an altogether separate, novel approach to the same problem. Here we list six key departures from the Sequence Logo which allow Sequence Bundles to overcome main limitations and weaknesses of the Logo:

**A -- **Shifting the focus of the visualisation from being position-oriented to sequence-oriented by explicitly maintaining continuity and integrity of each plotted sequence;

Reason: Residues' functions are associated with their position in relation to one another within proteins. Because Sequence Logos represent residues in isolation without valuable contextual information, their position-oriented focus limits their uses. The Sequence Bundles method is sequence-focused, therefore it allows to view a string of residues holistically as a functional protein, as well as to expose correlations and motifs, potentially assisting discovery (see the 'Results' section for examples).

**B -- **Using semi-opaque curved paths instead of deformed letters;

Reason: Deformed type is hard to read and stacking letters means that highly conserved ones rest on an uneven bed of less conserved ones, which makes them difficult to compare. Unfortunate stacking can also lead to letter misinterpretation (e.g. V above I in position 23 of the contest Logo could be misread as Y). Representing sequences with curved paths allows for their equal and proportional display with strong focus on sequence continuity. Atypical sequences are never removed but are faint enough to be inconspicuous.

**C -- **Reassigning the Y-axis from displaying the amount of information measured in bits to displaying letter-coded amino acids arranged by physiochemical properties;

Reason: We found that many bioinformaticians were uninterested in the level of detail about mutual information shown in protein alignments. For the purpose of protein conformation research, residue physiochemical properties are reportedly a far more important measure and deserve more refined and structured representation than crude colour-coding used in Sequence Logos (this also allows the Sequence Bundles method to adhere to the best practice of design accessibility for users with colour vision deficiency).

**Figure 2:**
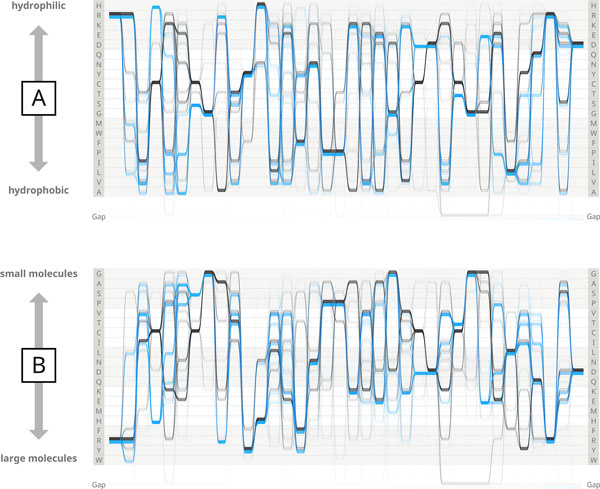
**Comparison of two Sequence Bundles plots differentiated by the Y-axis organisation**. Reorganising the Y-axis by different principles enables a more in-depth exploration of visualised data and assists in finding meaningful links between data alignment and the physical properties of amino acids displayed in the sequence. Panel A shows the Sequence Bundles visualisation of the AKL domain with the Y-axis organised according to amino acid hydrophobicity (hydrophilic to hydrophobic residues arranged top to bottom after Wampler [12]). Panel B shows the Sequence Bundles visualisation of the same dataset with the Y-axis organised according to amino acid molecule weight (small to large molecules arranged top to bottom after Lide [14]).

A comparison of two different Y-axis arrangements by amino acid physiochemical properties and their effects on the Sequence Bundles plots is shown in Figure 2 (ordering of amino acids by molecular weight after Lide [14]).

**D -- **Integrating three separate contest Sequence Logo figures into one combined visualisation, where both Gram-positive and Gram-negative bacteria can be directly juxtaposed;

Reason: It is very difficult to compare stacked letters across separate Sequence Logo figures, and we found that users frequently misjudged letters' height and relative proportions. By placing the two datasets on the same graph and differentiating by colour, the Sequence Bundles method enables an easy and direct comparison of both groups, whilst also offering a general overview of the whole population. Thus, any arbitrary collection of sets of sequences can be readily compared by stacking a given number of lines in Sequence Bundles, with each sub-Bundle remaining in direct visual relationship with the rest. The compound plot allows both overall and relative features to be observed, easily compared and contrasted.

**E -- **Visualising MSA gaps as a separate unit on the Y-axis;

Reason: MSAs rely on gaps to optimise alignment. Gaps are never shown in Sequence Logos, which dissociates visual representations from visualised data (although some Sequence Logo modifications visualise information about sequence insertions and deletions included in the alignment). Sequence Bundles displays gap locations within each sequence alongside gaps' actual length.

**F -- **Visualising explicitly all sequences included in the alignment and providing the total number of sequences in each colour group;

Reason: We discovered that scientists are often hesitant to trust Sequence Logos as they give no indication of the total number of compared sequences and proportions of sequences distributed between juxtaposed coloured groups. Logos generated from 9 or 9,000 sequences can look the same, but their credibility would be very different. To make this information sufficiently explicit and avoid visual clutter, transparency level applied to plotted lines is balanced against the total number of all visualised sequences.

## Results

### Current developments

The Sequence Bundles method has been implemented as a demonstration application written in the open source Processing language [15]. This application includes algorithms and methods responsible for visual encoding of already structured and formatted databases. In the visualisation pipeline outlined by Ward et al. [16], Sequence Bundles facilitates the 'Data to Visual Mapping' process and to some extent also the 'View Transformation' process. It does not support the 'Data Modelling' process or the 'Data Selection' process--these need to be completed outside of the Sequence Bundles demonstration application.

At this stage of development the Sequence Bundles demonstration application offers automated means of plotting and visually encoding a large number of sequences organised in a previously curated MSA. The Sequence Bundles demonstration application accepts input of sequences in plain text (TXT) file formats (including gaps). Two output types are supported: bitmap and vector graphics files. Bitmaps can be exported from the Processing default image renderer in a specified resolution. Vector graphics files can be exported to Portable Document File format (PDF) with preserved editing capabilities, measurements and scale, as well as specified colour and transparency settings (this is attained via an open source PDF Export library for Processing).

Using the Sequence Bundles demonstration application we have managed to discover a number of interesting features in the contest dataset, which are outlined below.

### Data features identified with sequence bundles

**Figure 3:**
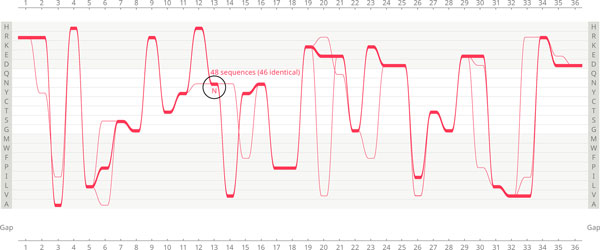
**Feature 1: Extreme conservation of sequences displaying asparagine in Gram-negatives in position 13**. Highlighting all AKL domain sequences in Gram-negative bacteria displaying asparagine (N) in position 13 exposes extreme conservation of the selected sequences throughout the length of the visualised protein. The total number of highlighted sequences is 48, including 46 identical (minor variation occurs only in two sequences in positions 2, 3, 6, 12, 14, 15, 20, 21, 23, 26, 30, 33 and 35). The underlying causes for this unusual conservation remain to be investigated. Due to the scale of this extreme conservation (nearly 5.4% of the whole Gram-negative dataset), the findings can have significant implications for the interpretation and evaluation of data.

**Figure 4:**
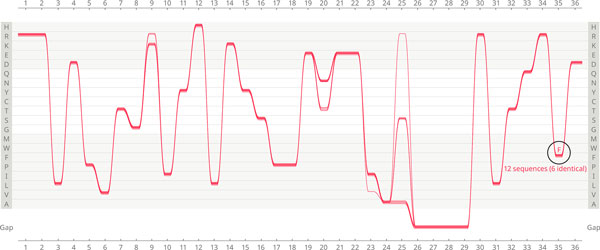
**Feature 2: Extreme conservation of sequences displaying phenylalanine in Gram-positives in position 35**. Highlighting all AKL domain sequences in Gram-positive bacteria displaying phenylalanine (F) in position 35 reveals that all of these sequences are extremely conserved throughout the whole length of the visualised protein with rare variation in positions 9, 20, 23 and 25. The total number of sequences in this selection is 12, out of which 6 are identical). The underlying causes for this unusual conservation remain to be investigated. One of possible reasons could be a mis-curation of the MSA (which requires to be confirmed).

**Figure 5:**
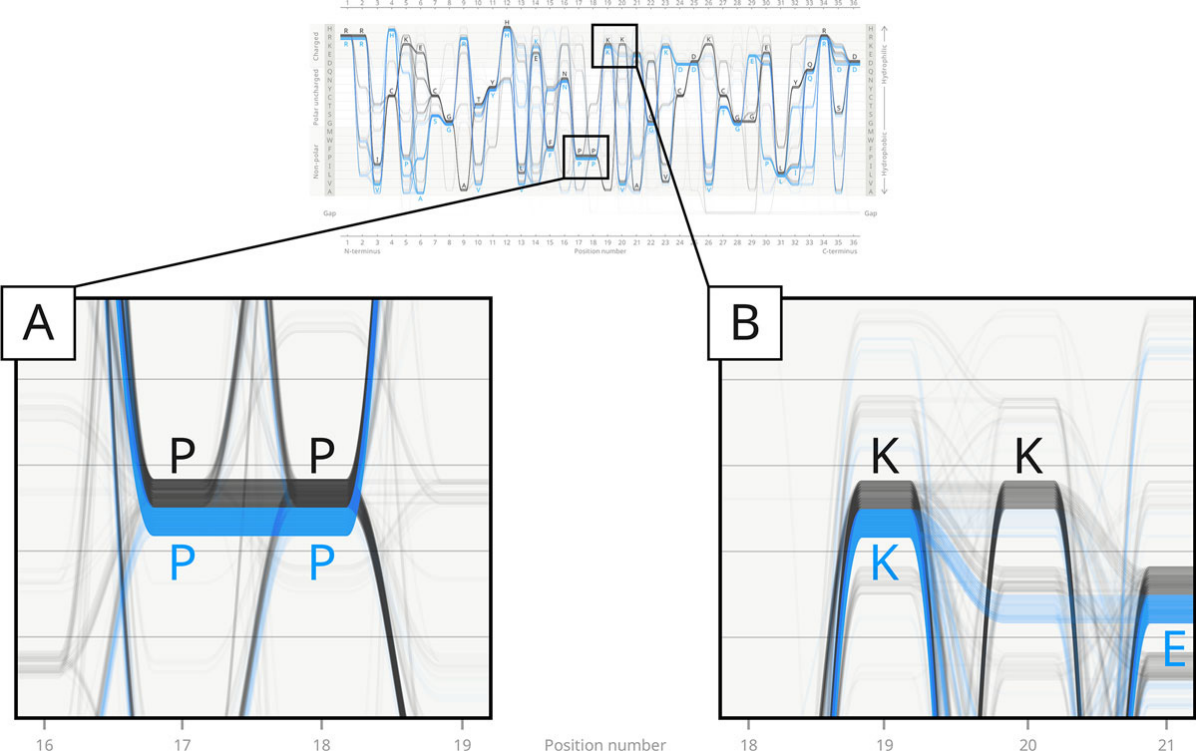
**Feature 3: Dissimilarity of distribution of two pairs of prolines and lysines in positions 17-20**. Close inspection of the AKL domain visualised with Sequence Bundles exposes the dissimilar nature of the distribution of two pairs of residues in the Gram-positives (black) sub-dataset, which is difficult to be observed with the use of existing visualisation methods or in the general consensus sequence (in positions 17-20 it is: ...PPKK...). The Sequence Bundles method preserves continuity of sequences by visualising them as uninterrupted lines which reveals that while the majority of sequences in positions 17-18 display a consecutive pair of prolines (indicated by a thick horizontal 'bridge' between P-P in panel A), one part of the Gram-positive sequences display a lysine in position 19, while another part display a lysine in position 20. Note that very few black lines bridge the gap between K-K in the Sequence Bundles visualisation (panel B)--the majority of sequences include only one of the lysines. This data feature remains hidden in the Sequence Logo, as well as in the general consensus sequence itself. In fact, only 23 sequences display the exact ...PPKK... motif fully consistent with the general consensus sequence. The reason for this dissimilarity of residue distribution in the MSA remains to be explained and interpreted.

**Figure 6:**
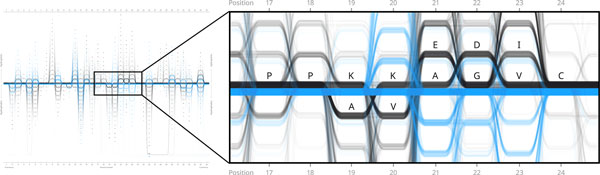
**Feature 4: Bifurcation of the general consensus sequence in the 'streamgraph' variant of the visualisation**. Restructuring the Sequence Bundles chart into a 'streamgraph' visualisation exposes the pattern of variation formed by sequences displaying most frequent residues in positions from 19 to 23 in Gram-positive bacteria (black). Note two interweaving threads bifurcating in position 19 and converging in position 24, one displaying: ...KVEGI... and the other: ...AKADV... Neither of these two parallel threads adhere to the general consensus sequence in Gram-positives which in positions 19-23 is: ...KKAGV... Connection 'bridges' between consensus residues in Gram-positive in positions 19-20, 21-22 and 22-23 are much less significant compared to strongly pronounced interweaving links between other frequent residues. This data feature can be saliently exposed owing to the fact that the Sequence Bundles visualisation method displays sequences as continuous lines and not as discrete items of statistical data in each position (as in the case of the Sequence Logo).

The development of the Sequence Bundles visual encoding and the demonstration application for generating vector visualisations enabled the exploration of the competition dataset in a novel visual manner. Various actions, such as rendering of the data according to different residue ordering principles (Figure 2.), brushing (i.e. making a selection by dragging the mouse cursor in an interactive visualisation view [17]) and highlighting of selected regions or close-up examination of interesting sections of sequences, led to the discovery of a number of interesting and potentially insightful features of the AKL domain dataset. In Figures 3, 4, 5, 6 we illustrate four of those features, specify details about each of them in figure legends and outline the methods by which they became exposed.

## Conclusions

We have created a novel visualisation method for displaying MSAs called Sequence Bundles and developed it as a demonstration application running in Processing. We have demonstrated the efficacy of our design decisions and the value of Sequence Bundles presentation of data by exposing a number of interesting and surprising features in the contest dataset, which would otherwise have remained hidden. Although it remains to be confirmed what scientific meaning the observed features have, the ability of the Sequence Bundles method to identify features in data that are of interest to the data authors themselves demonstrates the intuitiveness and high suitability of Sequence Bundles for visual exploration of MSAs.

The results of our visual investigation into the hidden patterns in the contest data also demonstrate the predisposition of the Sequence Bundles method for prospective implementation as a dynamic and interactive software tool for MSA visualisation and visual analysis. Conventional controls such as updatable rendering, zooming in and out, panning, colour-coding, as well as partitioning and splicing of datasets--currently attainable only through edits to the demonstration application code--would immediately streamline the process of exploration of the visualisation. Additional features such as additive and subtractive brushing, highlighting, annotating and toggling of axes would enable considerable flexibility, introduce instant user feedback and improve the general workflow. These are currently facilitated by taking advantage of vector graphics software user interface. In the longer perspective, software tools employing the Sequence Bundles method may benefit from introducing independent localised Y-axis organisation (as opposed to the prevalent global arrangement), smart algorithms to optimise disentanglement of the lines, or 3-dimensional presentation of data. Development of visual analytics programmes which would take full advantage of the Sequence Bundles visual encoding, would complement existing MSA visualisation tools well. This would not only increase the efficiency and scope of the bioinformatics workflow, but also open the bioinformatics domain for access by collaborators from other fields, as well as for interested non-experts.

## List of abbreviations

AKL -- adenylate kinase lid; EMBL-EBI -- European Molecular Biology Laboratory, European Bioinformatics Institute; MSA -- Multiple Sequence Alignment; MSAs -- Multiple Sequence Alignments (plural); PDF -- Portable Document Format; pHMMs -- profile Hidden Markov Models;

## Competing interests

Authors declare that they have no competing interests.

## Authors' contributions

MK and JK conceived the Sequence Bundles project as a submission to the BioVis 2013 redesign contest. MK acted as project coordinator and lead designer. MK developed the complete code for Sequence Bundles demonstration application and is responsible for exposing all data features presented. JK oversaw the team, managed the project and provided design direction. LN acted as the researcher for the project and wrote the contest submission paper. NG and RS acted as scientific consultants, proposed new features and gave feedback on preliminary versions of Sequence Bundles. All authors contributed to team meetings, discussions and workshops. MK wrote this manuscript, and JK, LN, NG and RS helped to draft it. All authors read and approved the final version of the manuscript.

## Authors' Information

MK, JK and LN work at Science Practice--a design consultancy with strong focus on collaborations with biological sciences and bio-medical industry.

MK is an information designer with strong interest in data visualisation and visual analytics. He lectured at universities in Europe and USA. He also authored and led tutorials in visual communication at 2012 and 2013 IEEEVis conferences.

JK is an interaction designer, the principal and the founder of Science Practice. He lectured at universities in Europe, USA and Asia. He is also one of the winners of the 2008 iGEM grand prix.

LN is an anthropologist and a developer. She is reading for an MSc in digital anthropology at UCL, London.

NG and RS are scientists at European Molecular Biology Laboratory, European Bioinformatics Institute (EMBL-EBI). NG is the leader of the Goldman research group at EMBL-EBI. The group's interest is in development of new models and data analysis methods for the study of molecular sequence evolution.

RS is a post-doctoral fellow in the Goldman group at EMBL-EBI.

## Declarations

Publication of this work was supported by Science Practice and EMBL.

This article has been published as part of *BMC Proceedings *Volume 8 Supplement 2, 2014: Proceedings of the 3rd Annual Symposium on Biological Data Visualization: Data Analysis and Redesign Contests. The full contents of the supplement are available online at http://www.biomedcentral.com/bmcproc/supplements/8/S2
